# Data on quantitation of *Bacillus cereus sensu lato* biofilms by microtiter plate biofilm formation assay

**DOI:** 10.1016/j.dib.2019.104951

**Published:** 2019-12-05

**Authors:** Rener De Jesus, Gina Dedeles

**Affiliations:** a The Graduate School, University of Santo Tomas, España, Manila 1008, Philippines; b Department of Biological Sciences, College of Science, University of Santo Tomas, España, Manila 1008, Philippines; c Research Center for the Natural and Applied Sciences, Thomas Aquinas Research Complex, University of Santo Tomas, España, Manila 1008, Philippines

**Keywords:** Microbiology, 1% Crystal violet solution, 96-Well microtiter plate, Absorbance microtiter plate reader

## Abstract

The microtiter plate biofilm formation assay is a method for the study of early biofilm formation on abiotic surfaces. It is a colorimetric technique that uses dyes, such as crystal violet, to stain attached biofilms and to quantify by using an absorbance microtiter plate reader. In this data, we evaluated the ability of 12 *Bacillus cereus sensu lato* isolated from soil and milk powder samples for their production of biofilms after a total of 48 hr incubation period in the 96-well microtiter plate. The biofilm production was induced by initially exposing them in diluted tryptic soy broth at its first 24 hr and then replacing with freshly prepared double strength broth for the next incubation period at 30 °C. The optical densities of the bacterial growth in the wells were read at the absorbance wavelength of 630 nm while the stained biofilms that solubilized in absolute ethanol were read at 570 nm. The biofilm measurements were calculated and the degree of biofilm production of each isolate was classified according to biofilm formation categories adapted from previous researchers. Therefore, the assay concluded the negative impact of *B. cereus* group by ability to form biofilms on abiotic surfaces, such as food contact surfaces in food production industries and the wide application of the current methods in research and industrial fields.

**Table:** Specifications Table

Subject	Microbiology
Specific subject area	Applied and food microbiology
Type of data	Text (absorbance wavelengths readings) Table
How data were acquired	Absorbance microplate reader (SH-1000 Lab, Corona Electric Co. Ltd., Japan)
Data format	Raw Analyzed
Parameters for data collection	The biofilm formation of *B. cereus s.l.* isolates in the 96-well microtiter plate wells were induced with nutrient-less tryptic soy broth [1:20(v:v)] in the first 24 hr incubation period at 30 °C. For the next 24 hr, the each well was supplemented with nutrient-rich TSB (double-strength) at the same incubation condition.
Description of data collection	Using an absorbance microtiter plate reader, the *B. cereus s.l.* growth in wells were read at 630 nm then the wells with crystal violet-stained biofilms attached on the surface, which solubilized by absolute ethanol, were read at 570 nm. The assay was performed at triplicate for each *B. cereus s.l.* isolate and the specific biofilm formation index was calculated to determine the biofilm formation category.
Data accessibility	The data on conventional identification of *B. cereus s.l.* isolates from soil and milk powder samples are available in Mendeley Data with DOI:10.17632/832 g5m5yvb.1, https://data.mendeley.com/datasets/832g5m5yvb/1

**Table:** 

**Value of the Data** • *B. cereus s.l.* biofilms have negative impacts, such as product cross-contamination, in food production industry. Therefore, these data can be used for evaluation of *B. cereus s.l.* biofilm formation on abiotic surfaces. • In general, biofilms can be studied in many fields, from medical to food and quality microbiology, thus researchers in these fields can benefit from the data presented here and support related studies. • Data shown here can be useful for initial evaluation of biofilm production by *B. cereus s.l.* on abiotic surfaces (e.g. microtiter plate well surfaces). The data will help various food production industries, especially in dairy manufacturing where *B. cereus* group is prominent, for the study of biofilms. • The procedure, with some modification, used here for the gathering of data can be applied to future research in relation to microbial biofilm quantitation.

## 1 Data

The microtiter plate (MTP), also known as 96-well plate, assay is a method for studying biofilms that are adhered on abiotic surfaces [1]. For quantitation of biofilms, the MTP assay is done using optically clear flat-bottom plates for measuring optical densities (ODs) by an absorbance MTP reader and the adhered biofilms are stained with crystal violet (CV).

In this method, the MTP assay was performed in three sets and in triplicates for each *B. cereus s.l.* isolate in every set. The raw data of conventional bacterial identification of 12 *B. cereus s.l.* isolates from soil and milk powder samples are available in Mendeley Data with https://doi.org/10.17632/832g5m5yvb.1. All ODs datasets that were read at absorbance wavelengths of 570 nm and 630 nm are presented in Table 1 and Table 2, respectively. Specifically, in Table 1 presents ODs at 570 nm of attached *B. cereus s.l.* biofilms on well surfaces and were stained with 1% CV solution while Table 2 shows ODs at 630 nm of bacterial growth in MTP wells after a total of 48 hr incubation period with TSB medium.

**Table 1:** MTP biofilm formation assay: optical densities of biofilms produced by *B. cereus s.l.* isolates after a total of 48 hr incubation in 30 °C read at 570 nm.

	Controls	*B. cereus s.l.* isolates											
		B-01	B-02	B-04	B-06	B-07	B-MS01	B-03	B-MS04	B-05	B-08	B-09	B-10
Set 1	1.064	2.196	1.490	1.425	2.200	1.503	1.483	1.534	1.568	1.715	1.422	1.540	2.195
	1.043	1.951	1.394	1.330	1.932	1.425	1.358	1.673	1.510	1.760	1.467	1.584	2.118
	1.052	1.970	1.270	1.297	2.132	1.320	1.566	1.555	1.478	1.600	1.497	1.675	2.056
Mean	1.053	2.039	1.385	1.351	2.088	1.416	1.469	1.587	1.519	1.692	1.462	1.600	2.123
SD	0.011	0.136	0.110	0.066	0.139	0.092	0.105	0.075	0.046	0.083	0.058	0.069	0.070
CoV (%)	1.0	6.7	8.0	4.9	6.7	6.5	7.1	4.7	3.0	4.9	3.1	4.3	3.3
Set 2	1.042	2.156	1.380	1.456	2.157	1.567	1.376	1.450	1.518	1.622	1.432	1.534	2.178
	1.073	2.230	1.450	1.306	2.006	1.480	1.430	1.511	1.576	1.533	1.562	1.598	1.965
	1.020	1.945	1.356	1.290	1.987	1.335	1.498	1.567	1.582	1.432	1.398	1.653	2.123
Mean	1.045	2.110	1.395	1.351	2.050	1.461	1.435	1.509	1.559	1.529	1.464	1.595	2.089
SD	0.027	0.148	0.049	0.092	0.093	0.117	0.061	0.059	0.035	0.095	0.087	0.060	0.111
CoV (%)	2.5	7.0	3.5	6.8	4.5	8.0	4.3	3.9	2.3	6.2	6.0	3.7	5.3
Set 3	1.013	2.003	1.390	1.387	2.116	1.496	1.558	1.493	1.483	1.593	1.569	1.783	2.187
	0.997	1.970	1.378	1.225	1.978	1.345	1.483	1.598	1.552	1.653	1.453	1.543	2.130
	1.056	1.854	1.498	1.360	2.067	1.277	1.323	1.602	1.468	1.513	1.388	1.598	1.993
Mean	1.022	1.942	1.422	1.324	2.054	1.373	1.455	1.564	1.501	1.586	1.470	1.641	2.103
SD	0.031	0.078	0.066	0.087	0.070	0.112	0.120	0.062	0.045	0.070	0.092	0.126	0.100
CoV (%)	3.0	4.0	4.6	6.6	3.4	8.2	8.3	4.0	3.0	4.4	6.2	7.7	4.7

**Table 2:** MTP biofilm formation assay: optical densities of cell growth by *B. cereus s.l.* isolates after a total of 48 hr incubation in 30 °C read at 630 nm.

	*B. cereus s.l*. isolates											
	B-01	B-02	B-04	B-06	B-07	B-MS01	B-03	B-MS04	B-05	B-08	B-09	B-10
Set 1	0.523	0.450	0.513	0.395	0.586	0.640	0.574	0.586	0.779	0.612	0.783	0.393
	0.580	0.468	0.601	0.418	0.535	0.576	0.568	0.518	0.853	0.546	0.824	0.431
	0.577	0.430	0.586	0.459	0.598	0.629	0.545	0.540	0.841	0.618	0.848	0.446
Mean	0.560	0.449	0.567	0.424	0.573	0.615	0.562	0.548	0.824	0.592	0.818	0.423
SD	0.032	0.019	0.047	0.032	0.033	0.034	0.015	0.035	0.040	0.040	0.033	0.027
CV (%)	5.7	4.2	8.3	7.6	5.8	5.6	2.7	6.3	4.8	6.7	4.0	6.5
Set 2	0.645	0.474	0.663	0.408	0.635	0.526	0.413	0.572	0.682	0.548	0.754	0.433
	0.619	0.519	0.598	0.420	0.606	0.576	0.483	0.540	0.698	0.615	0.811	0.417
	0.670	0.492	0.636	0.451	0.627	0.503	0.434	0.497	0.647	0.647	0.794	0.390
Mean	0.645	0.495	0.632	0.451	0.623	0.535	0.443	0.536	0.676	0.603	0.786	0.413
SD	0.026	0.023	0.033	0.022	0.015	0.037	0.036	0.038	0.026	0.051	0.029	0.022
CV (%)	4.0	4.6	5.2	5.2	2.4	7.0	8.1	7.0	3.9	8.4	3.7	5.3
Set 3	0.535	0.509	0.639	0.429	0.576	0.512	0.509	0.522	0.740	0.588	0.890	0.383
	0.639	0.461	0.598	0.452	0.539	0.593	0.451	0.530	0.820	0.637	0.787	0.419
	0.579	0.477	0.589	0.395	0.559	0.533	0.494	0.608	0.786	0.616	0.836	0.400
Mean	0.584	0.482	0.609	0.425	0.558	0.546	0.485	0.553	0.782	0.614	0.838	0.401
SD	0.052	0.024	0.027	0.029	0.019	0.042	0.030	0.048	0.040	0.025	0.052	0.018
CV (%)	8.9	5.1	4.4	6.7	3.3	7.7	6.2	8.6	5.1	4.0	6.2	4.5

The specific biofilm formation (SBF) index from each set of assay was calculated and the mean SBF indices are shown in Table 3. Furthermore, Table 3 also shows the sample sources from which the *B. cereus s.l.* test strains were isolated and their assigned biofilm formation categories (BFCs) according to Ref. [2].

**Table 3:** Microtiter plate biofilm formation assay: specific biofilm formation (SBF) index of *B. cereus s.l.* isolates and their biofilm formation categories.

Isolates	Sources	SBF index			mean SBF index ± SD^b^	CoV^c^ (%)	Biofilm Formation Category^a^
		Set 1	Set 2	Set 3			
B-01	soil	1.761	1.651	1.575	1.662 ± 0.094	5.6	+++
B-02	soil	0.739	0.707	0.830	0.759 ± 0.064	8.4	++
B-04	soil	0.526	0.484	0.496	0.502 ± 0.022	4.3	++
B-06	soil	2.441	2.228	2.428	2.366 ± 0.119	5.0	+++
B-07	soil	0.634	0.668	0.629	0.644 ± 0.021	3.3	++
B-MS01	milk powder	0.676	0.729	0.793	0.733 ± 0.059	8.0	++
B-03	milk powder	0.950	1.047	1.118	1.038 ± 0.084	8.1	+++
B-MS04	milk powder	0.850	0.959	0.866	0.893 ± 0.059	6.6	++
B-05	milk powder	0.775	0.716	0.721	0.737 ± 0.033	4.4	++
B-08	milk powder	0.691	0.695	0.730	0.705 ± 0.021	3.0	++
B-09	milk powder	0.669	0.700	0.739	0.703 ± 0.035	5.0	++
B-10	milk powder	2.534	2.528	2.696	2.586 ± 0.095	3.7	+++

a **+**, weak biofilm producer; **++**, moderate biofilm producer; and **+++,** strong biofilm producer.

b SD, standard deviation.

c CoV, coefficient of variation.

## 2 Experimental design, materials, and methods

### 2.1 Bacterial isolates

All *B. cereus s.l.* test organisms used in this study were isolated from soil samples and infant formula milk powders by the spread plate method in Ref. [3] using the *Bacillus cereus* selective agar® (Oxoid) supplemented with egg yolk emulsion and polymyxin B (Oxoid). A total of 12 *B. cereus s.l.* isolates were subjected to MTP biofilm formation assay. All isolates are kept in cryovials containing tryptic soy broth (Merck) with glycerol and stored at −80 °C for further analysis.

### 2.2 Biofilm formation of B. cereus s.l. isolates in 96-wells MTP

Following the described method in Ref. [2] with some modifications, the *B. cereus s.l.* isolates (n = 12) were grown overnight in 10 mL sterile TSB tubes at 30 °C in the incubator (Memmert GmbH + Co. KG). The polystyrene 96-well MTP (Corning®) were filled with 200 μL of TSB diluted with deionized water [1:20(v:v)]. The wells were inoculated with 3 μL of the overnight *B. cereus s.l.* cultures and incubated at 30 °C without shaking for 24 hr. After incubation, the broth in MTP wells were removed by gentle pipetting, replenished with freshly prepared sterile double-strength TSB to support the biofilm formation and incubated again at 30 °C for 24 hr.

### 2.3 Quantification of biofilm production

The growth in MTP wells was read at wavelength of 630 nm using an absorbance MTP reader as shown in Table 2. The broth of each well was removed and the wells were gently washed once with 200 μL sterile PBS, pH 7.2 and then air-dried for 20 min. The attached biofilms in the wells were stained with 130 μL 1% CV (LabChem) solution for 5 min and washed thrice with 200 μL sterile distilled water. The stained attached biofilms in the wells were solubilized in 130 μL absolute ethanol (J.T Baker®) and the ODs were read at 570 nm, which are presented in Table 1.

### 2.4 Specific biofilm formation and biofilm formation category

The biofilm quantities produced by the *B. cereus s.l.* isolates were calculated using the formula provided by previous researchers in Ref. [4], which is *SBF* index=(*AB*-*CW*)/*G*. The *SBF* stands for specific biofilm formation while *AB* is the OD read at 570 nm then *CW* is the OD of stained control wells containing un-inoculated medium (to eliminate unspecific or abiotic OD values) that read only at 570 nm, and *G* is OD of cell growth in broth measured at 630 nm. The assay for control wells was performed in triplicates. The mean and standard deviation of the *SBF* index were calculated and the degree of biofilm production was classified in three categories as described in Ref. [2] and presented in Table 3: weak (SBF < 0.5), moderate (0.5–1.0), and strong (SBF > 1).

### 2.5 Statistical treatment

The precision and repeatability of mean ODs reading and SBF indices in the MTP assay were determined using the coefficient of variation (CoV) below 10% as shown in Table 1, Table 2, Table 3
